# Chinese botanical drugs targeting mitophagy to alleviate diabetic kidney disease, a comprehensive review

**DOI:** 10.3389/fphar.2024.1360179

**Published:** 2024-05-13

**Authors:** Leilei Ma, Jing Li, Xiaotian Zhang, Wei Zhang, Chen Jiang, Bo Yang, Hongtao Yang

**Affiliations:** Department of Nephrology, First Teaching Hospital of Tianjin University of Traditional Chinese Medicine, National Clinical Research Center for Chinese Medicine Acupuncture and Moxibustion, Tianjin, China

**Keywords:** mitochondria, mitophagy, diabetic kidney disease, Chinese botanical drugs, treatment

## Abstract

Diabetic kidney disease (DKD) is one of the chronic microvascular complications caused by diabetes, which is characterized by persistent albuminuria and/or progressive decline of estimated glomerular filtration rate (eGFR), and has been the major cause of dialysis around the world. At present, although the treatments for DKD including lifestyle modification, glycemic control and even using of Sodium-glucose cotransporter 2 (SGLT2) inhibitors can relieve kidney damage caused to a certain extent, there is still a lack of effective treatment schemes that can prevent DKD progressing to ESRD. It is urgent to find new complementary and effective therapeutic agents. Growing animal researches have shown that mitophagy makes a great difference to the pathogenesis of DKD, therefore, exploration of new drugs that target the restoration of mitophagy maybe a potential perspective treatment for DKD. The use of Chinese botanical drugs (CBD) has been identified to be an effective treatment option for DKD. There is growing concern on the molecular mechanism of CBD for treatment of DKD by regulating mitophagy. In this review, we highlight the current findings regarding the function of mitophagy in the pathological damages and progression of DKD and summarize the contributions of CBD that ameliorate renal injuries in DKD by interfering with mitophagy, which will help us further explain the mechanism of CBD in treatment for DKD and explore potential therapeutic strategies for DKD.

## 1 Introduction

Diabetic kidney disease (DKD) is one of the serious microvascular complication in diabetic mellitus (DM), and approximately 40% of T2DM patients will develop to DKD (Stephens et al., 2020). The global prevalence of diabetes in the 20-79 age group is projected to rise to 12.2% by 2045, affecting an estimated 783 million people (Sun et al., 2022). DKD is a progressive disease characterized by the microalbuminuria in early stage, persistent massive proteinuria and increased creatinine levels in middle stage, and culminating in ESRD eventually. The renal pathology of DKD is mainly characterized by glomerular mesangial cell (GMCs) proliferation, glomerular basement membrane (GBM) thickening, and extracellular matrix accumulation, which eventually leads to renal fibrosis (Jiang et al., 2019; Wang et al., 2021). To date, few effective therapeutic strategies can inhibit the deterioration of DKD. The present strategies for treating DKD are still limited to strictly management of hyperglycemia, lipids, blood pressure, and the use of RASS blockers. Although more and more clinical trials have shown that the SGLT2 inhibitors will be a potential oral agents for prevent DKD (Mosenzon et al., 2019; Heerspink et al., 2020; Giglio et al., 2023). The non-steroidal selective mineralocorticoid receptor antagonist (MRA) finerenone has also been gradually proved to have great potential in the treatment of DKD (Filippatos et al., 2021; Filippatos et al., 2022; Agarwal et al., 2022). Renal replacement treatment (RRT) or kidney transplantation are still be the ultimate choice for DKD patients with ESRD. Therefore, it is necessary to further get a thorough understanding of the pathogenesis of DKD to explore new drugs including complementary and alternative medicine to delay the progression of DKD.

As we know, the kidney is the second highest oxygen consumption organ in our body. The kidney not only contains different cell types but also performs a variety of physiological functions such as endocrine functions, regulating blood pressure and intraglomerular hemodynamics, transporting solutes and water, maintaining acid-base balance, reabsorbing nutrients, and eliminating fuel or drug metabolites. The normal physiological function of kidney cells depends on adequate energy supply from mitochondria (Murphy and Hartley, 2018). However, more and more researches have shown that mitochodrial damages and dysfunction played an essential role in the pathophysiology of different kidney diseases, as well as in DKD (Ratliff et al., 2016; Duann and Lin, 2017). Numerous researches have shown that the dysfunction of mitochondria was involved in the accelerated progression of DKD (Wang et al., 2012; Coughlan et al., 2016; Long et al., 2016; Mise et al., 2020). Therefore, appropriate and timely removal of aged or abnormal mitochondria to maintain mitochondrial homeostasis is crucial to relieve the damage of DKD. Mitophagy is a highly conserved mechanism for selective removal of dysfunctional and fragmented mitochondria via the autophagic machinery, which has been recognized as a pivotal mechanism for regulating mitochondrial quality and quantity control (Lemasters, 2005; Palikaras et al., 2018; Pickles et al., 2018) A growing body of studies have proven that mitophagy is impaired *in vivo* and *in vitro*, and the signaling pathways regulating mitophagy are inhibited (Higgins and Coughlan, 2014; Czajka et al., 2015; Chen et al., 2018). Enhancing the level of intracellular mitophagy has a significant renoprotective effect in DKD and supplementation with MitoQ, a mitochondrial antioxidant, was confirmed to protect against DKD through upregulating the levels of mitophagy via Nrf2/PTEN-induced putative kinase protein 1 (PINK1) (Tagawa et al., 2016; Xiao et al., 2017; Yang et al., 2019b). Therefore, both basic and clinical studies aimed to modulate or restore impaired mitophagy may provide innovative therapeutic strategy for DKD.

CBD has a history of thousands of years and was used to treat various diseases during the long development of Chinese nation. Also, CBD is popular in the world and widely used in more than 100 countries owing to its safety clinical efficacy. Currently, more and more studies have confirmed that traditional CBD has unique advantages and good clinical efficacy in delaying the progression of DKD, which has gradually aroused the interest of nephrologists (Tang et al., 2021; Zhang et al., 2022; Shen et al., 2024).

Over the past decades, the potential molecular mechanisms of CBD for the treatment of DKD have been researched extensively. Multiple studies have revealed that CBD can exert renoprotective action through regulating autophagy (Xu et al., 2017; Wang et al., 2018; Zhan et al., 2019), and even mitophagy (Hang et al., 2018; Wang X. et al., 2020). This review will sum up the molecular mechanism of mitophagy during the occurrence and progression of DKD. Furthermore, recent advances in preventing DKD via regulating mitophagy from the perspective of CBD will also be discussed.

## 2 Regulatory signaling pathways of mitophagy

**FIGURE 1:**
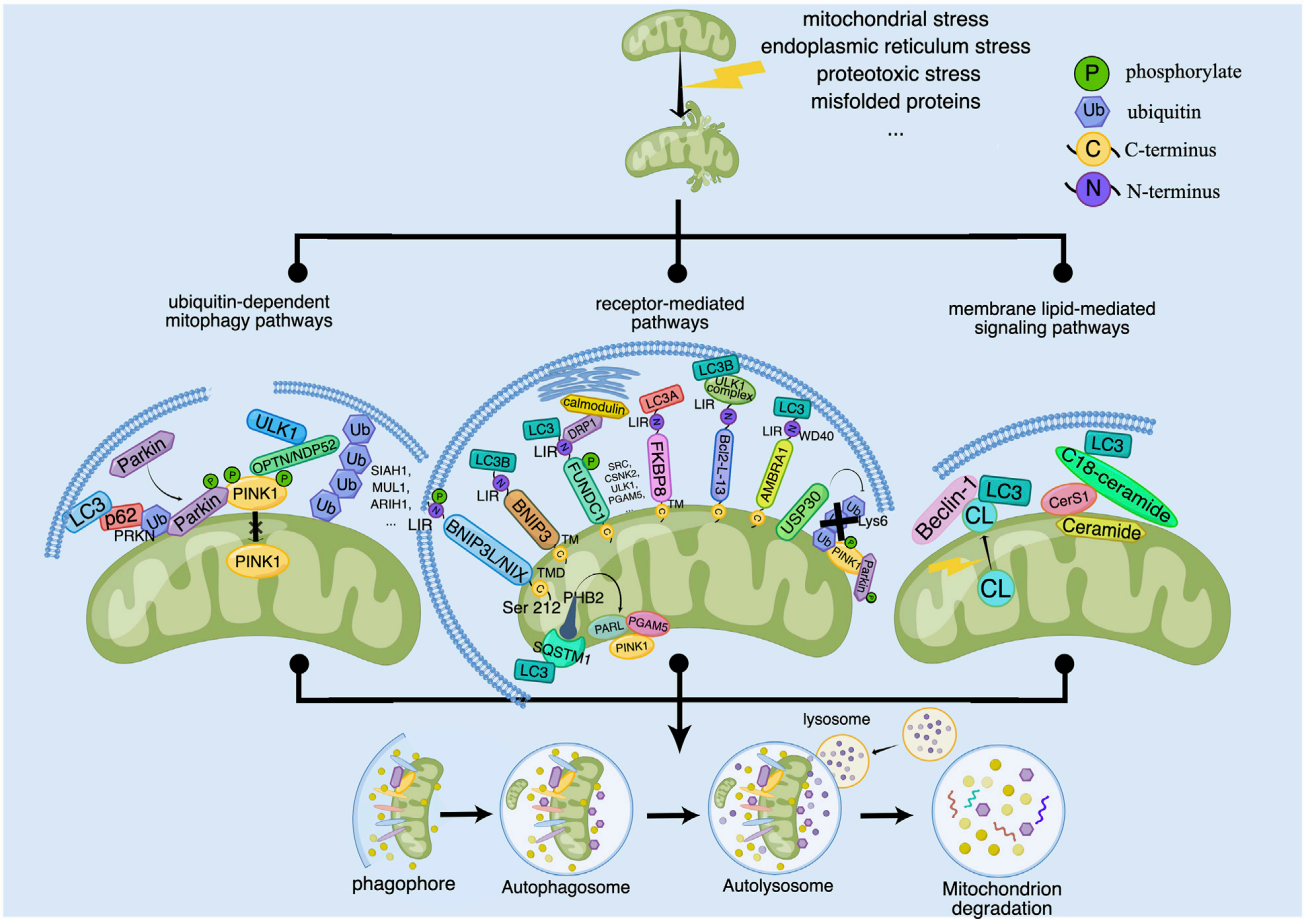
The signaling pathways for regulating of mitophagy. Some outer and inner risk factors such as Mitochondrial stress, endoplasmic reticulum stress, proteotoxic stress and misfolded proteins stimulate cells and lead to mitochondrial damage, which can trigger mitophagy to clear the damaged mitochondria. The main signaling pathways for regulation of mitophagy contain ubiquitin-dependent mitophagy pathways, receptor-mediated pathways and membrane lipid-mediated signaling pathways. Ub-dependent mitophagy involves the PINK1/Parkin dependent and non-PINK1/Parkin-dependent (receptor-mediated) pathways. PINK1 activates mitophagy by activating Parkin or recruiting OPTN/NDP52 and other ubiquitin ligases such as SIAH1, MUL1 and ARIH1. Receptor-mediated pathways involves the OMM and IMM proteins. OMM proteins include BNIP3L/NIX, BNIP3, FUNDC1, FKBP8, Bcl2-L-13, AMBRA1 and USP30, which regulate the coupling between OMM and the autophagosome membrane. IMM proteins include autophagy receptors such as PHB2. PHB2 can induce mitophagy by binding LC3 directly or forming a ternary protein complex with chelate 1 (SQSTM1) and LC3. In addition, PHB2 can regulate PINK1 by regulating the activity of mitochondrial protease PARL. Membrane lipid-mediated signaling pathways mainly include cardiolipin (CL) and ceramide. CL is located in the IMM in normal mitochondria. When mitochondria are damaged, CL is transferred to the OMM and interacts with LC3, which mediates mitophagy. And ceramide depends on CerS1 and C18-ceramide to promote the formation of LC3 and induce mitophagy.

Once fails to be repaired, mitochondria are eliminated by mitophagy to prevent excessive production of ROS and slow down apoptosis caused by inflammatory response damage. Typically, mitophagy is divided into PINK1/Parkin-dependent and independent pathways. Moreover, there are three principal pathways that modulate mitophagy in mammals: the Pink1/Parkin pathway, the BNIP3L pathway, and the FUNDC1 pathway. Among them, activation of PINK1, which is thought to be the initiating event for the induction of mitophagy, works vitally during the process of mitophagy. According to the available research results, the signaling pathways of regulating mitophagy are broadly divided into three categories: ubiquitin (ub)-dependent mitophagy pathways, receptor-mediated pathways and membrane lipid-mediated signaling pathways (Chourasia et al., 2015) (Figure 1).

### 2.1 Ubiquitination-dependent mitophagy pathway

#### 2.1.1 PINK1-Parkin pathway

At present, the PINK1-Parkin pathway has been most extensively studied. In the meantime, the most well-known protein components that control mitophagy for mitochondrial maintenance and quality control are PINK1 and Parkin (Pickles et al., 2018). PINK1 is a serine/threonine kinase located in the mitochondria whose function is an operator and sensor under the control of mitochondrial membrane potential (Zhou et al., 2008). In the wake of loss of mitochondrial membrane potential, PINK1 is stabilized and activated at the outer mitochondrial membrane (OMM). Located inside the cytoplasmic lysosome, parkin is an E3 ubiquitin ligase (Matsuda et al., 2010). Transfer of Parkin to mitochondria is essential for initiating mitophagy. When cells are stimulated, such as mitochondrial stress, proteotoxic stress and misfolded proteins, Parkin migrates to the OMM in large numbers, and works with ubiquitin-activating enzymes and ubiquitin molecules to ubiquitinize and modify damaged extramitochondrial membrane proteins, promoting the degradation of damaged mitochondria (Sarraf et al., 2013). Whereas, PINK1, located upstream of Parkin, exerts its functions to phosphorylate Parkin and ubiquitin, promoting the movement of Parkin from cytoplasm to the OMM (Narendra et al., 2010). Therefore, the activation of Parkin in response to mitochondrial damage is followed by ubiquitin phosphorylation of PINK1.

Normally, the PINK1 dimer remains stable until each kinase structural domain is phosphorylated (Gan et al., 2022). As the PINK1 protein translocates to the inner mitochondrial membrane (IMM), it is fragmented by PARL and then rapidly destroyed by ub-proteasome degradation (Sato and Sato, 2011). Once mitochondria are impaired or depolarized, PINK1 protein hydrolysis is inhibited and no longer degraded by cleavage, but rapidly accumulates in the OMM, where stable PINK1 is activated by autophosphorylation, and then recruits cytoplasmic Parkin to translocate to the surface of OMM, activating Parkin’s E3 ubiquitin ligase activity. Subsequently, receptor proteins, such as p62, recognize the mitochondrial ubiquitination signal and trigger the phosphorylation of activated PINK1 by kinase activity, then initiates the autophagic elimination of damaged mitochondria through LC3 and eventually enters the autophagic lysosomal pathway for degradation (Rakovic et al., 2011). Parkin and PINK1 act as a pair in degrading OMM proteins via ubiquitin-dependent degradation, which is required for mitochondrial surface protein renewal (Chan et al., 2011; Yoshii et al., 2011).

#### 2.1.2 PINK1 via Parkin non-dependent mechanism

According to general consensus, PINK1 stimulates mitochondrial protein ubiquitination by phosphorylating and activating Parkin (Koyano et al., 2014; Lazarou et al., 2015), but it can also promote mitochondrial ubiquitination independently of Parkin (Rojansky et al., 2016). Through its ubiquitin binding domain, PINK1 can recruit OPTN/NDP52 to mitochondria, where it induces ULK1 to trigger mitophagy (Lazarou et al., 2015). Moreover, it has been shown that during binding to PINK1, unmodified Ub can be converted to Ub-cr 88 conformation, stabilizing the complex and encouraging subsequent phosphorylation of Ser65 residues (Schubert et al., 2017). Additionally, other E3 ubiquitin ligases, such as SIAH1 and ARIH1, activate mitophagy in an independent manner from Parkin as well (Yao et al., 2021). They engage in the ubiquitination of mitochondrial surface proteins, followed by the recruitment of autophagic vesicles by OMM proteins to complete the phagocytosis and degradation of damaged mitochondria. In addition, TBK1 is capable of phosphorylating autophagy receptors, thereby enhancing the process.

### 2.2 Receptor-mediated mitophagy pathways

#### 2.2.1 Proteins in OMM

##### 2.2.1.1 Bcl-2/E1B- 19k-interacting protein 3-like receptor (BNIP3L/NIX)

BNIP3L/Nix, a uncharacteristic membership of the pro-apoptotic Bcl-2 subfamily of BH3 proteins, is also a protein embedded in the OMM through its c-terminal transmembrane structural domain (TMD) (Chinnadurai et al., 2008). It contains a WXXL modal structure (i.e.,LIR) next to its N-terminal end (Xie et al., 2020), which enables OMM to couple to autophagosomal membranes and also dialogue with autophagy regulatory mechanisms to induce mitophagy through its LIR motif (Hamacher-Brady and Brady, 2016). In contrast, its N-terminus can bind to the mTOR-activating protein Rheb, and thus the reduced effect of mTOR activation has an impact on autophagy enhancement (Li et al., 2007)). In mitophagy, the onset and development of BNIP3L requires a combined mechanism of LIR phosphorylation and BNIP3L dimerization (Marinković et al., 2021). BNIP3L dimerization is a potentially novel molecular mechanism. Serine 212 is the major amino acid residue at the C-terminus of BNIP3L that extends into the membrane space and is responsible for dimerization.

##### 2.2.1.2 Bcl-2/adenovirus E1B 19-kDa interacting protein 3 (BNIP3)

BNIP3, identical to BNIP3L, is a mitochondrial protein harboring a BH3 structural domain. BNIP3 has previously been determined to be a pro-apoptotic factor that interacts with adenovirus E1B-19 kDa and BCL2 proteins (Yasuda et al., 1998). BNIP3 is composed of a complex n-terminal region and a characteristic c-terminal transmembrane structural domain (Ogretmen and Hannun, 2004). Both BNIP3 and BNIP3L have the same N-terminal LIR, and their amino acid sequences are somewhat similar. At the same time, similar to Nix, BNIP3 regulates both mitophagy and cell death (Tang et al., 2019). As a key regulator of Parkin non-dependent mitophagy, BNIP3 binds to LC3B on autophagosomes and promotes phagocytosis of damaged mitochondria (Villa et al., 2018). Under hypoxic conditions, autophagy can be observed through the accumulation of the LC3 lipidated form (LC3II). Under the absence of BNIP3, mitophagy can be upheld by NIX, and elimination of pro-apoptotic mitochondria could contribute to the inhibition of apoptosis regulation (Abdrakhmanov et al., 2021) Nevertheless, other studies have shown that knockdown of BNIP3 resulted in increased residual mitochondria and increased expression of Nix on the membrane, but the damage caused by hypoxia was not compensated. Therefore, in BNIP3 knockout mice, an increase in Nix expression could be found, but the results also indicated that the level of mitophagy remained low and was uncompensated by the increase in Nix (Chourasia et al., 2015).

##### 2.2.1.3 FUN14 structural domain containing 1 receptor (FUNDC1)

FUNDC1 is a widely expressed protein localized to mitochondria and has been identified as a specific receptor for mitophagy under hypoxic conditions (Youle and Narendra, 2011). Moreover, research also revealed that FUNDC1 functions as a mitophagy receptor role in a carbonyl cyanide p-trifluoromethoxyphenylhydrazone (FCCP)-induced mitophagy (Liu et al., 2012). It covers three transmembrane regions of the OMM, one of which extending to the c-terminus of the mitochondrial intermembrane and one extending to the cytoplasmic n-terminus. FUNDC1 recruits LC3 via its LIR structure in the n-terminal region, and under normal conditions, FUNDC1 is unable to interact with LC3 due to Tyr18 and Ser13 phosphorylation. As a comparison, during hypoxia, Ser13 is dephosphorylated by the phosphatase PGAM5, assuring interaction between FUNDC1 and LC3 and thus starting mitophagy. Point mutations or deletions in the LIR structural domain results in impaired mitophagy mediated by FUNDC1 in HeLa cells. Interestingly, FUNDC1-mediated mitophagy is strongly influenced by FUNDC1 phosphorylation status (Lv et al., 2017). Analysis showed that after the interaction of Lys49 of LC3B with Ser17 of phosphorylated FUNDC1, the lateral chain of LC3B is subjected to a major rearrangement of structure to adapt to phosphorylated FUNDC1, thereby working as a sensor of the phosphorylation state of FUNDC1 (Kuang et al., 2016). The phosphorylation status of FUNDC1 is mediated by phosphatases like CSNK2 and SRC, which determines its interaction with Atg8 proteins to regulate mitochondrial turnover that is FUNDC1-induced (Terešak et al., 2022). Besides, FUNDC1 interacts not only with LC3B but also with DRP1 and calmodulin at the mitochondrial-endoplasmic reticulum junction, which seems to be required for the recruitment of DRP1, for the reason that mutant forms of FUNDC1 that cannot bind DRP1 unable to promote mitophagy.

##### 2.2.1.4 FK506 binding protein 8 (FKBP8)

FKBP8 (referred to FKBP38 as well) is a novel OMM mitophagy receptor belonging to the family of FK506 binding protein. The structural domain of FKBP8 is composed of four structural domains containing the n-terminal Glurich structural domain behind the peptidyl proline cis-trans isomerase structural domain, three tetrapeptide repeat structural domains, the calmodulin binding structural domain and a TM structural domain (Shirane-Kitsuji and Nakayama, 2014). FKBP8 is anchored in the OMM through its TM structural domain with the n-terminal pointing to the cytoplasm. Unlike other receptors, FKBP8 has a high affinity for LC3A and is significantly higher than LC3B. FKBP8 located in the OMM reacts with lipidated LC3A preferentially, interacts with lipidated LC3A in a LIR-dependent manner, and recruits LC3A to damaged mitochondria, ultimately causing mitophagy (Yoo et al., 2020; Aguilera et al., 2022). However, LC3A recruitment to mitochondria is not absolutely necessary, but the existence of FKBP8 does enhance LC3A recruitment to mitochondria, but particularly in response to mitochondrial stress (Bhujabal et al., 2017). What is interesting is that FKBP8 is not degraded by autophagic vesicles during this period (Vara-Perez et al., 2019). It has been found that FKBP8 escapes from the mitochondria to the endoplasmic reticulum in the course of CCCP-induced parkin-mediated mitophagy, where it binds to Bcl-2 to exert an anti-apoptotic effect and thus avoids mitochondrial autophagic degradation, which makes it essential for avoiding unnecessary apoptosis process of mitophagy (Saita et al., 2013). However, the mechanisms involved still deserve further investigation.

##### 2.2.1.5 Bcl-2-like protein 13 (Bcl2-L-13)

Bcl2-L-13 is a pro-apoptotic member of the BCL2 protein family, which is integrated into the OMM through its c-terminal transmembrane structural domain, while the N-terminal end is exposed in the cytoplasm. Likewise, Bcl2-L-13 is designated as a congener of autophagy-associated protein 32 (Atg32) in mammalian cells. Like Atg32, BCL2-L-13 has a mitochondrial localization and LIR motif (Murakawa et al., 2015). It is a mitochondrial receptor, which can make up for Atg32 in yeast (Mao et al., 2011). In general, mitochondrial depolarization trigger the expression of Bcl2-L-13, and knocking out Bcl2-L-13 prevents mitochondrial uncoupler carbonyl cyanide 3 chlorophenylhydrazone (CCCP)-induced mitophagy, whereas its overexpression is responsible for inducing mitochondrial disruption and mitophagy, but acts independently of DRP-1 and Parkin (Murakawa et al., 2015). Previous studies have identified the ULK1 complex, a counterpart of the Atg1 complex, as being required for Bcl2-L-13-mediated mitophagy in mammalian cells. After recruitment of the ULK1 complex, Bcl2-L-13 triggers mitophagy by means of the interaction of the LIR motif in the Bcl2-L-13-ULK1 complex with LC3B (Murakawa et al., 2019).

##### 2.2.1.6 Autophagy/beclin 1 regulatory factor 1 (AMBRA1)

AMBRA1 contains three motifs, including two PxP motifs, two TQT motifs and one LIR motif. AMBRA1 does not have an apparent structural domain, furthermore, only the WD40 structural domain, which contains about 40 amino acids and serves as a binding site for protein-protein or DNA interactions, exists at its N-termina (Maria Fimia et al., 2007). Thus, AMBRA1 can supply a framework for assembling protein complexes or mediating temporary interactions with other proteins (Jain and Pandey, 2018). As well, AMBRA1 has a significant role in apoptosis as an autophagy-associated protein and a direct substrate for cystathionin and calpain (Maria Fimia et al., 2007). During the stage of autophagy induction, AMBRA1 is capable of modulating ULK1 kinase activity, as well as interacting with BECLIN1 and VPS34 to manage relevant activities (Li X. et al., 2022). Under normal conditions, AMBRA1 has a preference to interact with mitochondrial BCL-2 (mito-BCL-2), and when mitophagy is activated, the interaction between AMBRA1 and mito-BCL-2 is broken (Strappazzon et al., 2011). It is found that AMBRA1 is a nonsubstrate interaction of Parkin, and the interaction between AMBRA1 and Parkin is strengthened after mitochondrial depolarization, resulting in mitochondrial removal in a Parkin-mediated way (Van Humbeeck et al., 2011). AMBRA1, a cofactor for E3 ubiquitin ligase HUWE1 activity, fosters the interaction of HUWE1 with MFN2, contributes to ubiquitination and degradation of MFN2, and impacts mitophagy eventually. On the other hand, HUWE1 promotes the interaction of the LIR motif of AMBRA1 with LC3 to induce mitophagy (Di Rita et al., 2018).

##### 2.2.1.7 Ubiquitin-specific protease 30 (USP30)

Constitutively, USP30 is the active deubiquitinase (DUB) known to be anchored in the MOM. Due to its unique transmembrane structural domain, it is found to be located in the OMM and peroxisome, and prefers to adopts a peculiar catalytic three-element and molecular construction to decompose the Lys6-linked ubiquitin chain prefavourably (Sato et al., 2017). Functionally, USP30 is a modulator of mitochondrial morphology and mitophagy (Yue et al., 2014). Acting upstream of PINK1 to set the threshold for initiating mitophagy (Marcassa et al., 2018), loss or reduction of USP30 levels leads to increased mitochondrial turnover (Bingol et al., 2014; Cunningham et al., 2015). USP30 is the only DUB related to mitochondrial surface composition, and it has been suggested that it mainly functions as an antagonist of mitophagy, either by removing ubiquitinated OMM proteins from Parkin substrates on the mitochondrial surface to antagonize PINK1/Parkin-mediated mitophagy (Bingol et al., 2014), or by reacting directly with Parkin to inhibit mitophagy (Wang Y. et al., 2020; Rusilowicz-Jones et al., 2020). The entire Lys6 link chain is prevented from hydrolyzing USP30 by phosphorylating distal ubiquitin by PINK1 (Wang et al., 2022), and ubiquitination of USP30 by Parkin may also contribute to its degradation, so Parkin and PINK1 may be able to moderate USP30. On the other hand, inhibition of USP30 expression or activity could allow cells to overcome the defects of PINK1 and Parkin, and restore the clearance of impaired mitochondria (Bingol et al., 2014). However, it has also been proven that USP30 can exert a regulatory effect on mitophagy independently of PINK1/Parkin activity under basal conditions (Rusilowicz-Jones et al., 2020).

#### 2.2.2 Proteins in IMM

##### 2.2.2.1 PHB2

PHB2, a member of the PHB family, contains an N-terminal transmembrane region essential for mitochondrial localization and also binds to PHB1 through the C-terminal loop region, participating as a complex to maintain mitochondrial structure and respiratory chain function (Osman et al., 2009). Moreover, PHB2 is also a highly conserved membrane scaffolding protein, and the presence of PHB2 on the IMM contributes to the maintenance of normal mitochondrial morphology, as well as the level of resistance to oxidative stress and apoptosis, which affects mitochondrial function (Artal-Sanz and Tavernarakis, 2010). On the one hand, PHB2 binds to the autophagosomal membrane-associated protein LC3 on damaged mitochondria through the LIR structural domain, and on the other hand, PHB2 combines with chelator 1 (SQSTM1) and LC3 to form a ternary protein complex in the loading of LC3 onto damaged mitochondria, which stimulated mitophagy (Xiao et al., 2018). PHB2 depletion destabilizes PINK1 in the mitochondria, preventing PRKN/Parkin, ubiquitin, and OPTN from being recruited to the mitochondria by the mitochondria after mitochondrial membrane depolarization or misfolded protein aggregation. By stabilizing PINK1 and increasing PRKN’s mitochondrial recruitment, PHB2 also encourages mitophagy through the PINK1/PRKN pathway (Yan et al., 2020). Significantly, we found that PHB2 regulates PINK1 processing by modulating the activity of the mitochondrial protease PARL. Furthermore, when mitochondria are depolarized, PHB2 stabilizes PINK1 via the PARL-PGAM5 axis.

### 2.3 Membrane lipid-mediated signaling pathways

#### 2.3.1 Cardiolipin (CL)

Cardiolipin (CL), also known as diphosphatidylglycerol, is produced by phosphatidylglycerol and cytidine diphosphate-diacylglycerol catalyzed by cardiolipin synthase (CLS) (Schlattner et al., 2014). Being a distinctive phospholipid of IMM, CL takes part in the cross-talk between lipid-protein and serves as part of the ingredients necessary for the maintenance of mitochondrial action (Khalifat et al., 2011). Redistribution of CL may also come into play in phagocytosis formation through binding to Beclin-1 and LC3 (Li X.-X. et al., 2015), and is likely to occur through autophagy protein interactions at sites of contact within and outside the membrane. It was reported that the distribution of CL could be interchangeable between the IMM and OMM in response to autophagic or apoptotic stimuli. In normal mitochondria, CL is located in the IMM. When mitochondria are damaged, a large portion of CL is transferred to the OMM and interacts with the autophagy protein LC3 to mediate mitophagy (Chu et al., 2014).

#### 2.3.2 Ceramide

Ceramide is a bioactive sphingolipid with particular structure. It is made of sphingosine backbone, and is esterified into fatty acyl chains by an amide bond at carbon 3 (Ogretmen and Hannun, 2004). Variations in the length of the fatty acyl chain yield numerous distinct ceramides, such as C14- to C26- ceramides (Saddoughi and Ogretmen, 2013). Mitochondrial ceramide derives from neutral sphingomyelinase (N-SMase) in response to increased production of reactive oxygen species (ROS) (Ogretmen and Hannun, 2004). As a core molecule in sphingolipid metabolism, ceramide takes part in regulating autophagy on a wide range of levels, which includes the selection of targets for the autophagic process, the induction of lethal mitochondrial autophagy and the elimination of damaged mitochondria. Ceramide accumulation in mitochondria can induce ceramide stress-induced mitophagy and ATP production reduction. It has been reported that Ceramide-induced mitophagy is dependent on ceramide synthase 1 (CerS1) and its metabolite C18-ceramide. Moreover, CerS1 and C18-ceramide invokes non-apoptotic lethal mitophagy selectively. Ectopic expression of CerS1 or processing of C18-ceramide facilitates the formation of LC3-II and its direct integration of ceramide and membranes of mitochondrial, attracting the binding of autophagosomes to damaged mitochondria and the occurrence of mitophagy (Sentelle et al., 2012). Some studies have shown that ceramides play a crucial role in Pink1 related Parkinson’s disease. Ceramides have been found to accumulate in mitochondria and have a negative impact on mitochondrial function (most notably, ETC.) (Vos et al., 2021). In addition, in the absence of PINK1, accumulation of ceramides can cause ceramide-induced mitophagy, to compensate for the loss of PINK1 dependent mitophagy. Therefore, reducing ceramide levels may be one of the treatment strategies for PINK1 related PD (Vos et al., 2022).

In summary, there are still many unknown fields regarding the specific molecular mechanisms of lipid mediated mitophagy. CL and ceramides are different molecules that can regulate mitochondrial dysfunction through signal transduction and recruitment of autophagy mechanisms. However, their regulatory mechanisms are still unclear, and more research is needed to explain the molecular process of lipid mediated mitophagy.

## 3 Mitophagy in kidney cells of DKD

As we know, the glomerulus is the basic structural unit of the kidney for filtration function, and is mainly composed of podocytes, endothelial cells and mesangial cells. These 3 cell types depend on each other through complex biological processes to maintain the normal physiological activity of glomerulus. In addition, as one of the cells highly enriched in mitochondria, renal tubular epithelial cells (RTECs) are also very important in the progression of DKD. In recent years, although some exciting results have been achieved about the specific mechanism of mitophagy in renal intrinsic cells during the progression of DKD, we still need great efforts to further confirm these results. Here is an overview of the relationship between the mitophagy in kidney cells and DKD currently (TABLE 1).

**TABLE 1:** Regulation of mitophagy in renal cells of DKD.

Renal cells	Upstream gene proteins	Mitophagy receptor	Effects on mitophagy	Effects on DKD	References
**Podocytes**	FOXO1	PINK1	FOXO1 overexpression prevented the HG-induced downregulation of PINK1 mRNA levels	FOXO1 overexpression, to some degree, promoted the recovery of injured podocytes	Li et al. (2016)
	Sirt1	BNIP3	Sirt1 positively regulates CR-mediated enhancement of hypoxia-induced autophagy upstream of Bnip3	Sirt1 mRNA expression levels correlated negatively with serum cystatin C levels	Kume et al. (2010)
	Sirt6	AMPK	Sirt6 overexpression could effectively reduce podocyte apoptosis accompanied by AMPK phosphorylation.; upregulation of Sirt6 was demonstrated to significantly reduce mitochondrial superoxide content and cellular ROS production	Sirt6 overexpression attenuates HG-induced mitochondrial dysfunction and apoptosis via promoting AMPK phosphorylation	Fan et al. (2019)
**Renal tubular epithelial cells**	TIPE1	PHB2	The deficiency of TIPE1 could promote its proteasomal degradation by regulating the expression of PHB2, which could propel the mitophagy of RTECs	The deficiency of TIPE1 couldultimately slow down renal tubular cell injury and EMT	Liu et al. (2022)
	-	OPTN	OPTN silencing significantly inhibited HG-induced mitophagosome formation, and overexpression of OPTN relieved cellular senescence through promoting mitophagy	In clinical specimens, renal OPTN expression was gradually decreased with increased tubulointerstitial injury scores.OPTN expression also negatively correlated with serum creatinine levels, and positively correlated with eGFR	Chen et al. (2018)
	DUSP1	Parkin	Overexpression of DUSP1 reverses the decreased Parkin protein in mitochondria	DUSP1 plays a defensive role in the pathogenesis of DN by restoring Parkin-mediated mitophagy	Lu et al. (2021)
	TXNIP	mTOR	TXNIP siRNA restored tubular mitophagy through inhibition of the mTOR signaling pathway	Blockade of TXNIP could suppress the production of interstitial collagens and reduce renal interstitial fibrosis in DKD	Huang et al. (2016)
	Nrf2	PINK	Nrf2-mediate regulation of PINK transcription and ameliorating mitochondrial oxidative stress and aberrant mitochondrial dynamics	Transfection with Nrf2 siRNA or PINK siRNA in HK-2 cells exposed to HG conditions partially blocked the effects of mitoQ on mitophagy and tubular damage	Xiao et al. (2017)
	STING1	PINK1	Activation of STING1 can upregulate the expression of PINK1 in HK-2 cells	Activation of STING1/PINK1 pathway can alleviate the injuries of kidney tissues of HFD/STZ-induced diabetic mice and HK-2 cells cultured in HG.	Zhu et al. (2023)
**Glomerular mesangial cells**	EPO	PINK1/Parkin	EPO can promote the expression of PINK1/Parkin-mediated mitophagy-related genes	EPO could attenuate renal injury and reduce oxidative stress	Yi et al. (2022)
**Glomerular endothelial cells**	FGF13	Parkin	The bifunctional role of FGF13 deficiency in promoting mitophagy and inhibiting apoptosis through Parkin can shape mitochondrial homeostasis regulation	Endothelial-specific deletion of FGF13 potentially alleviates T2DN damage, while FGF13 overexpression has the opposite effects	Sun et al. (2023)
	Nrf2/ARE	PINK	The specific Nrf2 inhibitor ML385 could inhibit mitophagy, as revealed by the decreased protein levels of PINK and Parkin	Activation of Nrf2/ARE signaling may restore diabetic nephropathy induced mitochondrial dysfunction and impaired renal function	Sun et al. (2019)

Annotation: FOXO1, Forkhead transcription factor O1; SIRT1, sirtuin 1; Sirt6:sirtuin 6; TIPE1, tumor necrosis factor alpha-induced protein 8-like 1; DUSP1, dual-specificity protein phosphatase 1; TXNIP, siRNA:Thioredoxin interacting protein siRNA; Nrf2, NF-E2-related factor 2; STING1, stimulator of interferon genes; EPO, Erythropoietin; FGF13, Fibroblast growth factor 13; PINK1, PTEN-induced putative kinase 1; BNIP3, Bcl-2, 19-kDa interacting protein 3; AMPK, AMP-activated protein kinase; mTOR, Mechanistic target of rapamycin; PHB2, prohibitin 2; OPTN, optineurin; ARE:antioxidant response elements.

### 3.1 Mitophagy in podocytes

Podocytes are glomerular visceral epithelial cells. The dysfuntion of podocyte structure and function influence in the development of DKD, and podocyte homeostasis has been used as one of the therapeutic targets in DKD (Dai et al., 2017). Podocytes have a high degree of terminal differentiation, resulting in a poor ability to re-enter the cell cycle and an inability to re-proliferate usually, and therefore rely heavily on autophagy, which is also usually exhibited at high levels (Hartleben et al., 2010). Autophagy is one of the fundamental self-healing mechanisms that maintain podocyte function, and its self-healing properties are essential for cell differentiation and proliferation (Wang and Choi, 2014). Podocytes are rich in mitochondria, which are essential components of the cytoplasm of podocytes and are the main energy-supplying organelles. Mitophagy maintains cell and tissue metabolism and homeostasis by removing damaged organelles (Mizushima and Komatsu, 2011; Feng et al., 2014; Xiong and Zhou, 2019), therefore, enhancing the activity of podocyte autophagy is of major importance for maintaining podocyte homeostasis (Liu et al., 2018).

High Glucose (HG) has been proven to promote mitochondrial dysfunction and podocyte apoptosis by suppressing mitochondrial autophagic activity (Li et al., 2016). Additionally, HG, hypoxia and abnormal immune response induce imbalance of mitochondrial homeostasis, deficient autophagy, inflammation and oxidative stress in podocytes (Clark and Parikh, 2021). Sirtuins (SIRTs) have been found to function synergistically in promoting mitophagy in podocytes and can ameliorate podocyte injury and proteinuria (Aventaggiato et al., 2021). SIRTs are class III histone deacetylases that rely on nicotinamide adenine dinucleotides (NAD+) and are relevant to various cellular signaling pathways, including mitochondrial function as well as autophagy (Kume et al., 2010). Among them, in glomerular disease models in humans and animals with DKD, the expression of SIRT1 is inclined to go down in renal cells (Yacoub et al., 2014). In DKD, hyperglycemia exacerbates podocyte apoptosis by raising the production of advanced glycosylation end products (AGEs), which increases FOXO4 acetylation and inhibits SIRT1 expression (Chuang et al., 2011). The mice podocyte with Sirt1 knocked out appeared severe proteinuria and renal fibrosis with mitochondrial dysfunction (Chuang et al., 2014; Liu et al., 2014). SIRT3 also inhibits permeability transition pore opening in mitochondria and improves mitochondrial function and dynamics, thereby mediating mitophagy (Zhao W. et al., 2021). What’s more, overexpressed SIRT6 protected mitochondria via phosphorylation of AMPK in podocytes (Fan et al., 2019).

There are also many receptors participate in the mitophagy of podocytes. Li et a (Li J. et al., 2020) proved that Smad4 was increased in both diabitics and mouse podocytes, and Smad4 located to mitochondria affect glycolysis and oxidative phosphate in podocytes induced by HG, thus causing podocyte damage. Overexpressed FOXO1 could active PINK1/Parkin-dependent mitophagy, thereby eliminating abnormal mitochondria and ameliorating damage of podocyte in diabetic mice induced by streptozotocin (STZ), which suggested the vital role of FOXO1 in regulating podocyte mitophagy (Li et al., 2017). Moreover, it was shown that both hyperinsulinemia and hyperglycemia could inhibit the autophagic activity of podocytes by inducing excessive activation of the autophagy regulatory protein mTORC1 (Mizushima and Komatsu, 2011). Chen et al. (Chen et al., 2021) found that HG can inhibit autophagy by activating the Janus kinase/signal transducer and transcription signaling pathway in mice and podocytes (Jakhar et al., 2016). At the same time, oxide accumulation, ubiquitinated proteins and endoplasmic reticulum stress may damage mice podocytes easily when the autophagy-associated protein 5 (podocyte-specific protein) is in absence, which eventually lead to proteinuria (Hartleben et al., 2010).

### 3.2 Mitophagy in renal tubular epithelial cells

The autophagy in RTECs could influence in kidney hypertrophy and tissue injury, which finally result in the development of DKD (Ma et al., 2020). And increasing evidences shown that the flawed mitochondrial dynamics and the excessive oxidative stress in mitochondria are principal factors of renal tubular injury in DKD (Higgins and Coughlan, 2014). Besides, more and more researches have also found that mitophagy in renal tubular is closely connected with tubulointerstitial injury in DKD (Chen et al., 2018). Han et al. (Han et al., 2021) have observed that the activation of mitophagy in RTECs from diabetic mice is downregulated significantly and they have testified that the AMPK agonist metformin could ameliorated oxidative stress and interstitial fibrosis in kidney by activating the p-AMPK-Pink1-Parkin pathway in diabetic mice induced by high fat diet (HFD) and STZ. The study has shown that Sirt3 prevents cell injury through AMPK-mediated autophagy (Zhao et al., 2018; Wang et al., 2019) Furthermore, Sirt3 also inhibits mitochondrial damage and cardiomyocyte apoptosis by activating autophagy and mitophagy in cardiomyocytes stimulated by HG (Yu et al., 2017). On this basis, Wang et al. (Wang Y. et al., 2021) further certified that HG environment could activite the Notch-1/Hes-1 pathway in RTECs, and the promotion of autophagy by Sirt3 was diminished after the activation of Notch-1/Hes-1 pathway. Therefore, it was suggested that Sirt3 may stimulate autophagy in RTECs by inhibiting the Notch-1/Hes-1 signaling pathway to finally achieve the therapeutic effect of DKD.

In addition, TIPE1, a novel partner of the tumor necrosis factor-α -induced protein 8 family, was initially supposed to be a potential molecule involved in cell necrosis and apoptosis, which may regulate the progression of cell death. Liu et al. (Liu et al., 2022) observed that TIPE1 was upregulated in RTECs from patients and mice with DKD, which in turn disrupted HG-induced mitochondrial homeostasis in RTECs via impairing the mitophagy mediated by PINK1/Parkin signaling pathway. Moreover, the deficiency of TIPE1 could promote its proteasomal degradation by regulating the expression of PHB2, which could propel the mitophagy of RTECs, ultimately slowing down renal tubular cell injury and EMT. Hence, we speculate that TIPE1 may take part in the underlying mechanisms of DKD and inhibit the progression of mitophagy in RTECs.

To remove damaged mitochondria, Opineurin (OPTN) is enrolled into mitochondria by PINK1 during the course of mitophagy. It has been found that, the regulators of mitochondrial autophagosome formation, PINK1 and OPTN, are significantly reduced after high glucose stimulation. Silencing OPTN notably inhibites HG-induced mitochondrial autophagosome formation, while overexpression of OPTN alleviates cellular senescence by promoting mitophagy. Moreover, according to the study of clinical specimens, with the increase of renal tubular interstitial injury score, renal OPTN expression gradually decreased. Therefore, OPTN-mediated mitophagy is crucial for regulating HG-induced senescence of RTECs in DKD (Chen et al., 2018).

Dual specific protein phosphatase 1 (DUSP1) is reduced in human proximal tubular epithelial (HK-2) cells under high-glucose conditions. In HK-2 cells stimulated by HG, overexpression of DUSP1 reverses the decreased parkin protein in mitochondria. Conversely, but the deletion of parkin reverses the efficacy of overexpressed DUSP1 on mitophagy and apoptosis (Lu et al., 2021). Hyperglycemia upregulates the expression of thioredoxin-interacting protein, which leads to proximal tubular cell injury and inhibition of mitophagy by inhibiting BNIP3 expression and activating the mTOR signaling pathway in DKD patients (Huang et al., 2016). In a previous study, HK-2 cells were induced to undergo defective mitophagy, mitochondrial dysfunction and apoptosis, and reduced expression of PINK and Parkin in a high-glucose environment. However, these changes were reversed by mitoQ, a mitochondria-targeted antioxidant which protect DKD tubular injury by regulating mitophagy. MitoQ partially blocked mitophagy and renal tubular injury when Nrf2 siRNA or PINK siRNA were transfected under HG conditions (Xiao et al., 2017).

Stimulator of interferon response cGAMP interactor 1(STING1) is an evolutionarily conserved transmembrane protein that localizes to the endoplasmic reticulum (ER) membrane in immune and non-immune cells. A growing body of research supports that STING1 is emerging as a key regulator of autophagy (Hopfner and Hornung, 2020; Zhang et al., 2021; Jiménez-Loygorri and Boya, 2024). A recent study reported a potential association between STING1 and PINK1. The activation of STING1 could promote mitophagy by up-regulating PINK1 in mouse cardiomyocytes (wang 2020c). The latest study showed that the expression of STING1 and PINK1 was downregulated in the kidney tissues of HFD/STZ-induced diabetic mice and in HK-2 cells cultured in HG, accompanied by decreased mitophagy activity. Treating HK-2 cells with the STING1 activator mtDNA and STING1 pcDNA could enhance the levels of PINK1 and parkin, which can reduce HK-2 cells damages exposed to HG (Zhu et al., 2023), which may provide an innovative therapeutic basis for DKD treatment.

### 3.3 Mitophagy in glomerular mesangial cells

As a major part of the intraglomerular mesangial region, glomerular mesangial cells (GMCs) play a crucial part in carrying on the normal structure of glomerular capillaries and maintaining the homeostasis of mesangial matrix in kidney (Abboud, 2012). One of the histological features of DKD is matrix accumulation due to hypertrophy of GMCs (Haneda et al., 2003). It has been found that autophagy’s level is suppressed in GMCs under prolonged high concentration of glucose and the reduced autophagic activity is in connection with the expressive suppression of FBW7 in GMCs induced by HG (Kitada et al., 2011; Gao et al., 2019). FBW7, a substrate recognition component of SCF-type ubiquitin ligase complex, connects ubiquitin to the target proteins, which concerning cell growth, proliferation, differentiation and apoptosis. In addition, HG environment can also induce GMCs senescence directly by downregulating the expression of connexin43 (Zhang et al., 2006). Additionally, one of the PAQR family, AdipoQ receptor 3 (PAQR3), participate in various biological processes like autophagy, cholesterol homeostasis, tumorigenesis and energy metabolism. The downregulation of PAQR3 reversed HG-induced activated PI3K/AKT pathway significantly, which inhibited cell proliferation and ECM accumulation in human GMCs instead (Li H. et al., 2018). It has been proved that AGEs-induced ROS take a vital part in mitochondrial depolarization-mediated apoptosis of GMCs (Xu et al., 2016). In addition, endoplasmic reticulum (ER) stress is important in the regulation of autophagy (Chandrika et al., 2015; Jakhar et al., 2016). AGEs induce apoptosis and death in GMCs and ER stress is the upstream of autophagy in GMCs exposed to AGEs (Chiang et al., 2016). Autophagy may make positive contribution to the apoptosis induced by AGEs in GMCs.

Erythropoietin (EPO) is a glycoprotein hormone excreted largely by kidney and acts in regulating erythropoiesis. *In vitro* experiments, EPO could promote autophagic flow, attenuate mitochondrial dysfunction, increase mitochondrial ROS and reduce the level of apoptosis in GMCs treated with HG. Besides, EPO could also increase the level of mitophagy by upregulating the expression of PINK1 and Parkin proteins in GMCs. It was also demonstrated that EPO can attenuate renal injury by promoting the expression of PINK1/Parkin-mediated mitophagy-related genes in DKD mice (Yi et al., 2022). To sum up, EPO attenuated the damage of DKD via restoring mitophagy mediated by PINK1/Parkin pathway.

### 3.4 Mitophagy in glomerular endothelial cells

Glomerular endothelial cells (GECs) are well differentiated cells with window pores and charged luminal glycocalyx layer. They are conducive to the glomerular filtration barrier (GFB) functionally (Fogo and Kon, 2010; Haraldsson and Nyström, 2012). In type 2 diabetic patients, reduced window pores in GECs are associated with the increased level of proteinuria and reduced glomerular filtration function (Weil et al., 2012). Previous researches have proved that in glomerulosclerosis models and human with DKD, endothelial dysfunction holds the key to the course of glomerular disease and DKD (Sun et al., 2013).

Mitophagy maintains the integrity of GECs and the ultrastructure of podocytes to keep the endostasis of GFB. It has been discovered that the mitochondrial structure is abnormal in the GECs of DKD experimental mode (Qi et al., 2017). Lenoir et al. (Lenoir et al., 2015) assessed the features of autophagy in diabetic GECs by STZ-induced mice with the specific deletion of Atg5 in endothelial cell. Compared to controls group, the proteinuria in diabetic mice with endothelial-specific deletion of Atg5 is more severe. They found glomerular capillary dilation and endothelial damage in diabetic mice with deficient autophagy of endothelial cells. Furthermore, ultrastructural analysis revealed the presence of GECs cytoplasmic disorganization, vacuolization, and isolated cells (mostly endothelial cells) in the glomerular capillary lumen by the diabetic mice accompanied with endothelial-specific deletion of Atg5, which redouble confirmed that the autophagy of endothelial cells plays a renoprotective role in the course of DKD.

Previous researches have indicated that the GECs prolonged exposured to high glucose environment leading to an excess production of the superoxide derived by mitochondria and a persistent accumulation of oxide, which caused the cell dysfunction and promoted glomerular injury in turn. Further, it is the increased level of mitochondrial superoxide in GECs that leads to GECs’ dysfunction, and interestingly, it also exerts pathological effects on the adjacent podocytes (Casalena et al., 2020). Furthermore, HG can cause mitophagy deficiency, mitochondrial dysfunction and apoptosis in GECs, accompanied by decreased expression of PINK and parkin. CoQ10 can restore the expression, activity and nuclear translocation of Nrf2 in GECs cultured with HG, thus upregulating the expression of PINK and parkin, which suggest that CoQ10 act as a potent mitochondrial antioxidant and play a beneficial role in DKD by restoring mitophagy via the Nrf2/ARE signaling pathway (Sun et al., 2019).

Fibroblast growth factor 13 (FGF13) is a participator of the FGF homologous factors (FHFs) subfamily and is also an ancestral gene of the FGF family (Itoh and Ornitz, 2008; Sun et al., 2023) found that the expression of FGF13 was increased in GECs in DKD and the specific deletion of FGF13 in endothelial cell ameliorated the apoptpsis of HFD + STZ-induced glomerular cell, which suggest that the expression of FGF13 has a positive relation with T2DN. In the course of the experiment, FGF13 deficiency was observed to activate mitophagy and prevent apoptosis, then maintaining intra-mitochondrial homeostasis. It was finally demonstrated that FGF13 may exert a dual regulation of both promoting mitophagy and inhibiting apoptosis by affecting Parkin expression, ultimately exerting a crucial link for regulating mitochondrial homeostasis in the progression of DKD.

## 4 Chinese botanical drugs for treating DKD by regulating mitophagy

There are thousands of years in China that CBD has been used to solve clinical problems. Previously, studies have indicated that CBD alleviates blood glucose levels (Zhao M.-M. et al., 2021), peripheral neuropathy (Yu et al., 2021), peripheral vascular disease (Zhao et al., 2019) and other complications in diabetics. Some botanical drugs also show their renoprotection by reducing proteinuria and serum creatinine in DKD. And one of the mechanism is that CBD decrease inflammation, renal fibrosis, apoptosis and other pathological processes via the regulation of mitophagy (TABLE 2).

**TABLE 2:** Chinese Botanical Drugs in regulating mitophagy for treatment of DKD.

Types of CHM	Names of herbal medicine	Models	Target	Pathways	Ref. and year
Chinese botanical drugs decoction	Tangshen formula	db/db mice	PINK1↑,Parkin↑	PINK-1/Parkin signaling pathway	Chen et al. (2024)
	Huangqi-Danshen decoction	db/db mice	PINK1↓, Parkin↓	PINK1/Parkin pathway	Liu et al. (2020)
	Astragalus aksuensis Bge and Panax notoginseng F. H. Chen formula	HFD + HSD + STZ-induced rats	p-mTOR↓, PINK1↑, Parkin↑	mTOR/PINK1/Parkin pathways	Wen et al. (2020)
		HG-induced RMCs			
Single or couples of Botanical drugs	San-Huang-Yi-Shen Capsule	HFD + STZ-induced rats	PINK1↑, Parkin↑	PINK1/Parkin pathway	Song et al. (2018)
	Ginseng-Sanqi-Chuanxiong extracts	HG/PA-induced HAEC	AMPK↑	AMPK pathway	Wang et al. (2020a)
	Huangkui capsule	HFD + STZ-induced rats	STING1↑,PINK1↑,Parkin↑	STING1/PINK1 signaling pathway	Zhu et al. (2023)
		HK-2cells induced by HG			
Natural chemical metabolites	Esculetin	Type 1 diabetic rats with AKI and NRK-52E cells grown in HG exposed to sodium azide	Nrf2↑,Keap1↑,PINK1↑	Nrf2/PINK1/Parkin signaling pathway	Dagar N,et al. (2024)
	Icariin	STZ-induced rats	Sesn2↑, Keap1↓, Nrf2↑, HO-1↑, NLRP3↓	Sesn2/Keap1-Nrf2/HO-1/NLRP3 axis	Ding et al. (2022)
		HG-induced MPC-5 cells			
	Astragaloside II	STZ-induced rats	Keap1↓, Nrf2↑, PINK1↑, Parkin↑	Keap1/Nrf2-PINK1/Parkin pathway	Su et al. (2021)
	Dioscin	HFD + STZ-induced rats	PINK1↑, Parkin↑	PINK1/Parkin pathway	Tao et al. (2018)
	Jujuboside A	HFD + STZ-induced rats	CaMKK2↑, AMPK↑, p-mTOR↓, PINK1→, Parkin↑	CaMKK2-AMPK-p-mTOR and PINK1/Parkin pathways	Zhong et al. (2022b)
	Astragaloside IV	db/db mice	PINK1↓, Parkin↓, p-Parkin (Ser 65)↓, LC-3II↓	PINK1/Parkin pathway	Li et al. (2022a)

Annotation: PINK-1, PTEN-induced putative kinase 1; HFD, high-fat diet; HSD, high-sugar diet; STZ, streptozocin; RMCs, renal mesangial cells; p-mTOR, Phosphorylated mammalian target of rapamycin; HG/PA-induced HAEC, high glucose and palmitate-induced human aortic endothelial cell; AMPK, AMP-activated protein kinase; HK-2cells, Hexokinase 2; AKI, Acute Kidney Injury; NRK-52E, the kidney tubular epithelial cells; STING1, stimulator of interferon response cGAMP, interactor 1; Nrf2, NF-E2-related factor 2; Keap1, Kelch-like ECH-associated protein 1; HG, high glucose; MPC-5, cells, Mouse Podocyte Clone-5; Sesn2, Sestrin2; HO-1, Haem Oxygenase 1; NLRP3, Nucleotide-binding oligomerization domain (NOD)-like receptor protein-3; CaMKK2, calmodulin-dependent kinase 2; LC-3II:light chain 3.

### 4.1 Chinese botanical drugs decoction for regulating mitophagy in DKD

Tangshen formula (TSF) is a classic Chinese herbal formula used in China to treat DKD. It consists of Astragalus mongholicus Bunge [Fabaceae; astragali radix], *Centella asiatica* (L.) Urb. [Apiaceae; centellae herba], Stephania tetrandra S. Moore [Menispermaceae; stephaniae tetrandrae radix], Prunus persica (L.) Batsch [Rosaceae; persicae semen], Rheum palmatum L. [Polygonaceae; rhei radix et rhizoma]. Chen et al. found that TSF has the potential to inhibit DKD through the PINK-1/Parkin-mediated mitophagy process based on network pharmacology results. Subsequently the researchers conducted the vivo experiments to validate the pharmacology results. HFD-induced db/db mice were divided into three groups randomly: the model group, the low-dose TSF treatment group (6.79 g/kg/d), and the high-dose TSF treatment group (20.36 g/kg/d), which were fed continuously for 8 weeks. The results confirmed that TSF ameliorated kidney damage and restored mitochondrial structure in db/db mice and promote mitophagy by activating the PINK-1/Parkin signaling pathway (Chen et al., 2024).

Huangqi-Danshen decoction (HDD) is Chinese botanical drugs formula compound consisting of Astragalus mongholicus Bunge [Fabaceae; astragali radix] and Salvia miltiorrhiza Bunge [Lamiaceae; salviae miltiorrhizae radix et rhizoma], both of them have been used to improve anti-inflammatory and antioxidant (Li G.-H. et al., 2018; Ye et al., 2020). Xinhui Liu administered HDD (6.8 g/kg/day by gastric irrigation) to db/db mice for 12 weeks, then the mice treated with HDD maintained lower blood glucose and urinary ACR level. The renal histopathology revealed that HDD could alleviate glomerular hypertrophy, reduce the increase of mesangial cells and mesangial matrix, and slow down the extensive fusion of the visceral epithelial cells. Besides, the autophagosome encapsulating mitochondria were observed in renal tissue in db/db mice with transmission electron microscopy, but did not be found in HDD treatment group. The fewer expression of PINK1 and Parkin protein in HDD group suggested that HDD improved renal function by inhibiting instead of activating mitophagy mediated by PINK1/Parkin in db/db mice (Liu et al., 2020).

*Astragalus aksuensis Bge* and Panax notoginseng F. H. Chen formula (APF) is Chinese botanical drugs formula compound which is made up of Astragalus mongholicus Bunge [Fabaceae; astragali radix], Panax notoginseng (Burkill) F.H.Chen [Araliaceae; notoginseng radix et rhizoma], Angelica sinensis (Oliv.) Diels [Apiaceae; angelicae sinensis radix], Achyranthes bidentata Blume [Amaranthaceae; achyranthis bidentatae radix], Laminaria japonica Aresch. [Laminariaceae; Laminariae Thallus Eckloniae Thallus]. Using Agilent High performance liquid chromatography (HPLC) system combined with LC solution software and UV spectrophotometer to determine the five main effective metabolites of APF, namely, Astragaloside I, Astragaloside IV, Ferulic Acid, Calycosin, and β-ecdysterone. APF was used to solve the clinical problems in kidney diseases for several years. According to the research form Wen et al., the renal pathology in DKD mice showed the expansion of mesangial matrix and ECM and the fibrosis of tubule-interstitial, which could be ameliorated by the treatment of APF. The studies in autophagy deficiency of DKD (C57BL/6 mice induced by STZ + HFD) and cell (RMCs induced with HG) modle proved that APF could suppress p-mTOR and induce the activation of PINK1/Parkin signaling pathway. When they inhibited the autophagy of renal mesangial cells (RMCs) induced by HG with 3-methyladenine (3-MA), the benefit of APF was reduced. The result suggested that APF could delay the progression of DKD via enhancing the mitophagy level, which is mediated by mTOR/PINK1/Parkin pathway in RMCs (Wen et al., 2020).

### 4.2 Single or couples of botanical drugs for regulating mitophagy in DKD

San-Huang-Yi-Shen Capsule (SHYS) is composed of Astragalus mongholicus Bunge [Fabaceae; astragali radix], Panax quinquefolius L. [Araliaceae; panacis quinquefolii radix], Dioscorea oppositifolia L. [Dioscoreaceae; dioscoreae rhizoma], Tetradium ruticarpum (A.Juss.) T.G.Hartley [Rutaceae; euodiae fructus], Cuscuta australis R. Br. [Convolvulaceae; cuscutae semen], Polygonatum sibiricum Redouté [Asparagaceae; polygonati rhizoma], Rehmannia glutinosa (Gaertn.) DC. [Orobanchaceae; rehmanniae radix], Pogostemon cablin (Blanco) Benth. [Lamiaceae; pogostemonis herba], Hamamelis mollis Oliv. [Hamamelidaceae; hamamelis mollis Oliv. ], Leonurus japonicus Houtt. [Lamiaceae; leonuri fructus], Salvia miltiorrhiza Bunge [Lamiaceae; salviae miltiorrhizae radix et rhizoma],Conioselinum anthriscoides 'Chuanxiong’ [Apiaceae; chuanxiong rhizoma],Atractylodes macrocephala Koidz. [Asteraceae; atractylodis macrocephalae rhizoma], which has been demonstrated to improve renal function in diabetic patients (Song et al., 2018). Li et al. given SHYS(intragastric administration 0.81 g/kg and 1.62 g/kg for SHYS low-dose group and the high-dose group, respectively) to the rats induced by HFD and STZ for 8 weeks, and found that SHYS could protect renal function and reduce histopathologic changes in DKD rats. SHYS could improve the expression on Parkin, PINK1 and LC3-II protein and reduce the expression of p62, NLRP3, VDAC1, Tom20, and COXIV protein, which suggests that SHYS enhances the mitophagy mediated by Pink1/Parkin pathway and avoids mitochondrial and inflammatory injury to protect renal function from DKD (Li H. et al., 2022).

Another research demonstrated Ginseng-Sanqi-Chuanxiong (GSC) extracts (ferulic acid, notoginsenoside R1, ginsenoside Rg1, ginsenoside Re, and ginsenoside Rb1), which were extracted from Panax quinquefolius L. [Araliaceae; panacis quinquefolii radix], Panax notoginseng (Burkill) F.H.Chen [Araliaceae; notoginseng radix et rhizoma],Conioselinum anthriscoides 'Chuanxiong’ [Apiaceae; chuanxiong rhizoma], could improve the expression and activation on AMPK. Under high glucose and palmitate-stressed conditions, GSC enhanced mitophagy on human aortic endothelial cell via the regulation of AMPK pathway. To some extent, the research provides a new method of Chinese medicine to improve the damage of RTECs caused by DKD (Wang S. et al., 2020).

Huangkui capsule (HKC)is a traditional Chinese medicine prepared from the extract of Abelmoschus moschatus Medik. [Malvaceae; Abelmoschus moschatus Medicus], and its total flavonoids (TFA) are its main active chemical metabolites. In China, HKC was widely used to treat various types of kidney diseases, including DKD (Li P. et al., 2020; Zhao et al., 2022). HKC has been shown to improve the DKD of NOD mice by regulating the gut microbiota, and subsequently improved the levels of metabolites (Shi et al., 2023). Kim et al.demonstrated that HKC prevented the accumulation of renal pathogenic proteins and mitochondrial dysfunction by regulating autophagy and mitochondrial dynamics, thereby alleviating the progression of DKD (Kim et al., 2018). A recent study found that STING1/PINK1-mediated mitophagy is impaired in the kidney in a diabetes mouse model induced by HF-D combined with STZ. Treatment with a high dose of HKC (2.0 g/kg/day) ameliorated the renal tubular injury accompanied by upregulation of STING1/PINK1 signaling pathway mediated mitophagy and mitochondrial recovery. *In vitro*, HKC administration significantly protected the mitochondrial dynamics and function in HK2 cells. This effect was dependent on the activation of STING1/PINK1 signaling pathway by HKC to increase mitophagy (Zhu et al., 2023).

### 4.3 Natural chemical metabolites for regulating mitophagy in DKD

Esculetin, a naturally occurring hydroxycoumarin derivative found in plants and fruits of Citrus limonia, Cortex fraxini, and Fraxinus rhynchophylla. Some studies have shown that a range of pharmacological activities including ROS scavenging, anti-inflammatory, and antifibrotic action and protective effect of diabetes-associated chronic complications (Kadakol A., et al., 2015a; Kadakol A. et al., 2015b; Kadakol A., et al., 2016; Kadakol A., et al., 2017). Dagar et al. explore the potential effect of esculetin on IRI-AKI associated with diabetes by conducting different experiments. *In vivo*, T1DM Wistar rats were treated with two doses of esculetin (50 and 100 mg/kg/day orally) for 5 days followed by AKI established by bilateral ischemic-reperfusion injury (IRI). *In vitro*, esculetin (50 µM) treatment for 24 h was given to the NRK-52E cells grown in HG before which were exposed to sodium azide (10 mM) for induction of hypoxia/reperfusion injury (HRI). The results showed that treatment with esculetin significantly reduced the expression of specific markers for renal injury and increased the expression of PINK1 and Parkin. Besides, esculetin reduced mitochondrial oxidative stress by increasing the expression of Nrf2 and Keap1. Esculetin may alleviate mitochondrial dysfunction by inducing PINK1/Parkin mediated mitophagy, suggesting that esculetin is expected to become an effective therapy to prevent AKI-diabetes comorbidity (Dagar N,et al., 2024).

Icariin (ICA) is an essential constituent of flavonoid extracted from *Epimedium brevicornu Maxim.*[*Berberidaceae; epimedii folium*] (Li C. et al., 2015), which is a traditional Chinese herbal. It possesses numerous pharmacological functions such as ameliorating inflammation (El-Shitany and Eid, 2019), delaying renal fibrosis (Wang M. et al., 2021) and improving mitochondrial dysfunction. Qiao C et al. (Qiao et al., 2018) administered ICA(10uM) to HG-treated MPC-5 cells for 48hs. Then, the relevant biochemical indices and the ultrastructure of the podocytes revealed that ICA could reduce renal injury. And the increase in LC3, Sesn2, PINK1, PARK2 and mitophagy dye show that ICA could promote mitophagy in cultured cell and animal models. To figure out the principles of ICA in mitophagy, they inhibited the expression of Sesn2, Nrf2 and HO-1 respectively. It finally proved that under HG conditions, ICA could degradate Keap1 and activate Nrf2, and induce the activation of Nrf2/HO-1 signalling pathway to downregulate inflammatory cascade in connection with NLRP3, which was dependent on Sesn2-induced mitophagy (Ding et al., 2022).

Astragaloside II (AS-II) is a kind of saponin extracted from the root of Astragalus mongholicus Bunge [Fabaceae; astragali radix praeparata cummelle](Peng et al., 2008), which has anti-inflammatory (Wan et al., 2013) and immunoregulation (Qiao et al., 2019) effects in various diseases. The diabetic rat model was established by intraperitoneal injection of streptozotocin (STZ) at 55 mg/kg and treated with AS-II (3.2, 6.4 mg/kg/d) for 9 weeks to explore the protective effects of AS II on podocyte injury in DKD. The results indicated that AS II ameliorated albuminuria, renal histopathology, and podocyte foot process effacement and podocyte apoptosis in diabetic rats. Moreover, treated with AS-II also downregulated Keap1 protein level, upregulated Nrf2 expression, and promoted the expression of mitophagy-related protein PINK1 and Parkin in diabetic rats. In consequence, AS II ameliorates renal injury to the diabetic rats induced by STZ through the enhancing mitophagy of podocyte and the ability resistance to oxidative stress through regulation of Nrf2 and PINK1 (Su et al., 2021).

Dioscin is a steroidal saponin extracted from various kinds of vegetables and herbs belonging to the family of Dioscoreaceae, which shows the effect of anticancer, anti-infection, immunoregulation and hypolipidemic (Yang et al., 2019a). Dioscin reduced hyperglycemic and pancreatic injuries, and alleviated the markers’ level of renal function and the histopathology of kidney in HFD + STZ-induced rats by difference pathways (Tao et al., 2018). Zhong et al. found that dioscin (20 mg per kg bw) effectively reduced blood glucose, inflammatory factor expressions, pancreatic injury, renal function markers and renal pathological changes in DKD (induced by HFD and STZ) rat kidneys. Moreover, dioscin can also reverse the NOX4 expression and disorder of the mitochondrial respiratory chain. Mitophagy and mitochondrial fission/fusion were improved by dioscin via regulating Parkin, PINK1, DRP1, p-DRP1 and MFN2 expressions. The results suggested that the enhancement of mitophagy maybe one of the mechanisms of Dioscin in its renoprotective effect (Zhong et al., 2022a).

Jujuboside A (JuA) is a triterpenoid saponins extracted from Ziziphus jujuba Mill. [Rhamnaceae; ziziphi spinosae semen]and has a lot of beneficial functions, such as anticancer (Wang W. et al., 2021), oxidation resistance and anti-inflammatory (Wang et al., 2023). Zhong et al. studied the reno-protective effects of JuA on HFD and STZ induced DKD rats. JuA (20 mg/kg) and Metformin (Met, 300 mg/kg) were administrated to diabetic Sprague Dawley rat for 8 weeks daily. Compared with untreated group, fasting blood glucose and renal damage were significantly decreased after JuA treatment. Further studies certified that the renoprotection of JuA on DKD may be connected with the improvement in autophagy and mitophagy caused by the activation of CaMKK2-AMPK-p-mTOR pathways and Pink1/Parkin pathways (Zhong et al., 2022b).

Astragaloside IV (AS-IV) is one of the major and active metabolites of Astragalus membranaceus Bunge [Fabaceae; astragali radix praeparata cummelle]. Liu et al. (Liu et al., 2017) administered AS-IV(adding to standard feed at a dose of 1 g/kg) to the db/db mice for 12 weeks to explore the mechanism of AS-IV in the treatment of DKD. The result showed that AS-IV ameliorated renal injury evaluated by urinary albumin excretion (UAE) as well as renal pathology, which was independent of the decrease in blood glucose and body weight. Moreover, the expression of PINK1, Parkin, p-Parkin (Ser65) and LC-3II protein were abnormally increased in db/db mice and were downregulated by AS-IV. So they inferred that AS-IV retarded renal injury by inhibiting the mitophagy, which is mediated by PINK1/Parkin in db/db mice.

## 5 Conclusion and future perspective

Here, we have summarized the regulatory pathways of mitophagy and its potential role during the pathogenesis and progression of DKD, as well as the aspects of treating DKD by using CBD in the context of mitophagy regulations. Despite these encouraging findings, there are still many uncertain issues should be tackled in the further study. Firstly, in the physiology and pathology of DKD, moderate mitophagy can help to maintain mitochondrial homeostasis by removing senescent and necrotic mitochondria, but whether excessive activation or inhibition of mitophagy will further promote the progression of the disease remains unclear. Secondly, the kidney is a multicellular organ and different types of cells play specific roles in the progression of DKD. Therefore, the modulation and role of mitophagy in the kidney may be different in several renal innate cells, but the accurate regulation mechanism of mitophagy in various types of renal cells still need to be further explored. Thirdly, many signaling pathways and mechanisms, like oxidative stress, AGEs, inflammation, participate in the deterioration of DKD. Whether there is signal crosstalk between selective mitophagy and other signaling pathways also need to be investigated. Finally, although current studies have shown that the regulation of mitophagy may delay the progression of DKD, more efforts should to make to validate the preclinical findings in human samples and test the potential therapeutic implications in clinical trials. Moreover, how to precisely target mitophagy to treat DKD is also a great challenge in future.

Substantial evidence supports a significant role of CBD for treating DKD via regulating mitophagy, which shows us a glimmer of light to explore a promising therapeutic approach for ameliorating DKD. Despite these exciting results, there are still many unanswered include but not limited to the following questions to be addressed. In the first place, the current researches are more prone to focus on single or blended bioactive components derived from botanical drugs, which is not appropriate considering the benefits of multi-component and multi-pathway of CBD prescription. In another, the existing mechanisms of CBD in improving DKD by interfering with mitophagy are mainly focused on ubiquitin-dependent mitophagy pathways, special attention should be paid to receptor-mediated and membrane lipid-mediated signaling pathways. What is more, whether different traditional CBD have the same modulation of mitophagy is still a mystery. Last but not least, more studies should be conducted to clearly elucidate the kidney injury and beneficial function during the process of CBD against DKD targeting mitophagy. For example, Li et al. established an efficient method for the isolation of natural parkin ligands by centrifugal ultrafiltration and liquid chromatography/mass spectrometry. They successfully identified potential parkin ligands in Chinese medicine Polygoni Cuspidati Rhizoma et Radix and Sophorae Flavescentis Radix, and confirmed that there were 5 (kurarinol I, kurarinol one, kurarinol flavone G, Apigenin and emodin) could activate parkin in vitro self-ubiquitination assay, which provides the possibility of discovering more precise active components of CBD targeting mitophagy for the treatment of DKD in the future (Li et al., 2022). We believe that more and more mechanism studies will provide new potential treatment methods for DKD based on the perspective of mitophagy.
